# Pre-Diagnosis Diet and Physical Activity and Risk of Cardiovascular Disease Mortality among Female Cancer Survivors

**DOI:** 10.3390/cancers14133096

**Published:** 2022-06-23

**Authors:** Daphne Weikart, Dan Lin, Radha Dhingra, Laila Al-Shaar, Kathleen Sturgeon

**Affiliations:** 1 Department of Food Science, The Pennsylvania State University, University Park, PA 16802, USA; dkw81@psu.edu; 2 Department of Public Health Sciences, The Pennsylvania State University, Hershey, PA 17033, USA; dlin1@pennstatehealth.psu.edu (D.L.); rdhingra@phs.psu.edu (R.D.); lshaar83@gmail.com (L.A.-S.)

**Keywords:** Alternative Mediterranean Diet, metabolic equivalent task, retrospective cohort study, neoplasm, California Teachers Study

## Abstract

**Simple Summary:**

The number of cancer survivors is increasing; however, cancer survivors are at an increased risk of cardiovascular diseases (CVD) and CVD mortality. Therefore, it is pertinent to understand how lifestyle choices such as dietary patterns and physical activity are associated with this risk. Little is known about the relationship of pre-cancer diagnosis diet quality and physical activity (PA) with CVD among cancer survivors. Most studies have focused on post-cancer diagnosis risk factors, without accounting for their status in the pre-diagnosis period or interaction on CVD mortality. We examined pre-cancer diagnosis diet quality and physical activity in relation with CVD mortality risk in female cancer survivors from the California Teachers Study cohort. We hypothesized that higher diet quality scores and higher physical activity levels prior to cancer diagnosis would be associated with lower risk of CVD mortality in female cancer survivors.

**Abstract:**

Sub-optimal diet and physical activity (PA) levels have been associated with increased risk of cardiovascular disease (CVD) mortality. The relationship between pre-cancer diagnosis diet quality and PA level on CVD mortality risk in cancer survivors is unclear. We examined the association between pre-cancer diagnosis diet quality and leisure-time PA and their interaction on CVD mortality in cancer survivors. Diet quality was characterized by the Alternative Mediterranean Diet Index (aMED). Leisure-time PA was converted to a metabolic equivalent of task hours per week (MET-h/wk). During a median of 6.3 years of follow-up of 18,533 female cancer survivors, we identified 915 CVD deaths. aMED score was not associated with CVD mortality. PA level was inversely associated with CVD mortality (HR_Q1-Q4_ = 0.74; 95% CI: 0.61–0.88; P_trend_ = 0.0014). Compared to cancer survivors with the lowest pre-diagnosis aMED score and PA level, cancer survivors with higher aMED scores and higher MET-hrs/wk were at a 33% lower risk of CVD mortality (HR = 0.67; 95% CI: 0.52–0.87). Overall, this study shows PA to be a strong predictor of CVD mortality in female cancer survivors. Our observations support the importance of PA throughout the lifecycle in lowering CVD mortality risk.

## 1. Introduction

It is estimated that there are about 16.9 million cancer survivors in the US, and this number is expected to rise to over 20 million by 2030 [1]. With earlier detection of cancer and improved treatment protocols and therapies, the number of cancer survivors is increasing [2]. Cancer survivors face additional health consequences, as previous research has shown that cancer survivors are at a higher risk of cardiovascular disease (CVD) mortality compared to the general population [3,4]. In addition, heart disease is the leading cause of death among women in the US [5]. Women are less likely to receive preventive treatment or guidance in reducing risk factors than men at similar risk [6]. Women also tend to have less specific symptoms of heart disease than men, such as nausea, fatigue, or shortness of breath, making detection more difficult [7]. To understand the risk of CVD mortality, especially in an at-risk population such as female cancer survivors, it is important to understand the role of preventive lifestyle behaviors, such as diet and physical activity (PA) [8,9,10,11].

Poor diet and low PA levels are independently associated with higher risk for cancer and CVD mortality [8,9,10,11]. To prevent and manage chronic diseases, nutritional scientists have developed different indices to quantify a healthful diet. One such index is the Alternative Mediterranean Diet Index (aMED). Higher adherence to the Mediterranean diet includes higher intake of fish, fruits, vegetables, nuts, legumes, and whole grains instead of refined grains, and lower consumption of alcohol and red/processed meat. The Mediterranean dietary pattern has been associated with lower CV morbidity and mortality risks as well as improvement in CVD risk factors [12]. However, the literature examining the relationship between diet quality and CVD mortality in cancer survivors is scarce, as most of the literature has focused on cancer-specific and all-cause mortality outcomes [13,14,15,16,17,18,19,20,21,22]. With the increased risk of CVD in cancer survivors, it is important to know if this extends to CVD mortality.

Physical activity is also important in primary and secondary prevention of chronic diseases such as CVD. Higher PA levels have been associated with lower risk of CVD incidence and mortality [11]. The American Heart Association (AHA) and the American Cancer Society currently recommend adults get at least 7.5 MET-hrs/wk, preferably spread throughout the week [23,24]. Similar to studies assessing the role of diet in cancer, investigations of exercise and CVD risk in cancer survivors have focused on post-cancer diagnosis PA [25,26,27]. These studies did not address pre-cancer diagnosis PA or its change from before to after cancer diagnosis.

In this study, we investigated the association of pre-cancer diagnosis diet quality and PA level with risk of CVD mortality after cancer diagnosis. We hypothesized that a higher aMED score and higher PA level prior to cancer diagnosis would be associated with lower risk of CVD mortality in female cancer survivors. To our knowledge, no previous investigation has assessed pre-diagnosis diet quality and PA level or their interaction with CVD mortality in female survivors of all cancer types. The results of our study will inform primary care physicians and cardiologists as to the relative benefit a healthy lifestyle prior to a cancer diagnosis may have on risk for CVD mortality when determining prevention and treatment methods in cancer survivors.

## 2. Materials and Methods

### 2.1. Study Population

The California Teachers Study (CTS) is an ongoing prospective cohort study of 133,477 women that are active and retired public school teachers/administrators that completed a 16-page mailed questionnaire in 1995–1996 (questionnaire 1) [28]. This detailed questionnaire consisted of questions regarding possible risk factors for cancer including, but not limited to, body mass index (BMI), medications, diet, PA, alcohol intake, menopausal status, and smoking. Follow-up questionnaires were mailed in 1997–1998, 2000–2001, 2005–2006, 2012–2015 and, 2017–2019 for updates. Participants were included in the analysis if they had no history of cancer diagnosis, stroke or heart attack at baseline. Participants were excluded if they (1) had discrepancies that made their baseline questionnaires unusable, (2) did not live in California at baseline, (3) had missing variables in the food frequency questionnaire (FFQ) and total energy intake less than 600 kilocalories per day or above 3500 kilocalories per day, (4) had missing values for the leisure-time PA variable at baseline, or (5) were diagnosed with cancer one year following questionnaire 1. This secondary analysis of the CTS included 18,533 female cancer survivors. The use of this dataset for analysis was approved by the institutional review boards (IRBs) at The Pennsylvania State University (Hershey, PA, USA) and the City of Hope (Duarte, CA, USA).

### 2.2. Diet and Physical Activity Assessment

A detailed description of the dietary assessment has been published elsewhere [29]. Briefly, a 103-item, semiquantitative, validated FFQ [30] was completed as part of questionnaire 1 and questionnaire 4 to assess the dietary intake of participants in the past year. Participants indicated their frequency of consumption (categories ranged from never to once/day or 5+/day depending on the item) and portion size (small, medium or large relative to a given standard medium portion for questionnaire 1 and based on pictures using cup sizes for questionnaire 4). For computation of aMED for questionnaire 1, we used an index described previously [29] with nuts and legumes as separate categories. The aMED score for questionnaire 4 was calculated with the same food items that were consistent with questionnaire 1. For alcohol intake, ounces of alcohol consumed of each alcoholic beverage were calculated as the product of the reported intake based on specific drink size in ounces and the frequency of intake, multiplied by the respective percentage of alcohol included in each beverage (4.8% for beer; 12% for wine; 40% for liquor). The total amount was then multiplied by a factor of 28.3 to convert ounces/day to grams/day. A higher aMED score represents higher adherence to a Mediterranean diet, with the highest possible score being 9.

PA level was assessed based on questions regarding leisure-time PA level in questionnaire 1 and questionnaire 4. Participants reported whether they engaged in moderate exercises (e.g., brisk walking, golf, and volleyball) and strenuous exercises (e.g., swimming laps, aerobics, calisthenics, running, and jogging) in the past 3 years, and the frequency. Score of metabolic equivalent of task (MET) was assigned to standardize PA intensity using the 2011 Compendium of Physical Activities [31]. Moderate and strenuous exercise were assigned a score of 4.5 and 7.0 METs, respectively.

### 2.3. Outcomes

Cancer survivors were identified by using the date of diagnosis from any cancer, except non-melanoma skin cancer, through linkage of the CTS with the California Cancer Registry records [28,32], a mandated, statewide, population-based cancer reporting system. CVD mortality was defined based on the date-appropriate International Classification of Diseases (ICD), Ninth Revision codes 390–459 (through September 2015) or Tenth Revision codes I00-I99 (October 2015 and forward), through linkage with California mortality files, the Social Security Death Index, and the National Death Index records.

### 2.4. Statistical Analysis

Hazard ratios (HRs) and 95% confidence intervals (CIs) were estimated using multivariable Cox proportional hazards regression models with number of years since cancer diagnosis as the time scale. Person-time was calculated from the date of cancer diagnosis until (1) date of death, (2) they moved out of California for more than 4 consecutive months, or (3) 31 December 2017, whichever came first. aMED scores and PA MET-hours/week were ranked into quintiles and quartiles, respectively, with the lowest categories serving as the reference groups.

Baseline characteristics were standardized by age except age of participants and age at cancer diagnosis. Multivariable Cox regression models adjusted for the time interval from baseline to cancer diagnosis date and age at cancer diagnosis (model 1), in addition to race, smoking status, menopausal status and hormone use, total energy intake, and daily vitamin use (model 2). Further adjustments were made after including either PA for the analysis of diet quality (model 3) or aMED scores for the analysis of PA (model 4). To reduce potential bias from reverse causation and disease severity, sensitivity analyses were performed after restricting the analysis to female cancer survivors who had their dietary and PA data collected in questionnaire 4, at least one year after their cancer diagnosis date. These analyses of the association of pre-cancer diagnosis aMED scores and PA were adjusted for post-diagnosis aMED scores and PA, respectively. Due to the small number of cancer survivors with available data in both questionnaires 1 and 4, aMED scores and PA variables were analyzed as tertiles. Additionally, a post hoc power analysis was conducted.

Interaction between aMED score and PA level was assessed by using these two variables as indicator variables. Specifically, the -2log likelihood ratio (−2 LL) test was conducted to compare the differences between the full model with interaction terms and the reduced one without interaction terms. Trend tests were conducted by assigning median values for each category as a continuous variable. All statistical analyses were conducted in SAS 9.4 (Cary, NC, USA) in the CTS’s secure shared workspace [33]. Statistical significance was defined as a two-sided *p* value < 0.05.

## 3. Results

A total of 18,533 female cancer survivors were included in this study, of whom 40.9% had breast cancer, 10.6% melanoma of the skin, 6.6% uterine cancer, 5.5% lung cancer, and 36.4% another type of cancer. Table 1 shows the age-adjusted characteristics for the study population by quintiles of the aMED score. Participants with higher aMED scores were older, more physically active, less likely to be obese, had higher alcohol and total energy intake, had daily vitamin consumption, and were more likely to have a late-life cancer diagnosis. Participants with higher PA levels were associated with lower BMI, greater alcohol use, daily vitamin consumption, and were more likely to be white (Supplemental Table S1). These participants were also less likely to be smokers or have a history of diabetes or hypertension.

During a median time of 6.3 years of follow-up, we identified 915 CVD deaths. No significant association was observed between the aMED score and CVD mortality, as shown in Table 2. Pre-cancer diagnosis PA level was inversely associated with risk of CVD mortality, and the inverse association remained significant after the simultaneous adjustment for diet quality (Table 2; Model 4: P_trend_ = 0.0014). Compared to the lowest quartile, female cancer survivors with 13.5-<27.5 MET-hrs/wk of PA prior to cancer diagnosis had a 20% lower risk of CVD mortality (Model 4: HR_Q1-Q3_ = 0.80; 95% CI: 0.67–0.96). Female cancer survivors with pre-cancer diagnosis PA levels of ≥27.5 MET-hrs/wk also had lower risk of CVD mortality (Model 4: HR_Q1-Q4_ = 0.74; 95% CI:0.61, 0.88). 

**Figure 1:**
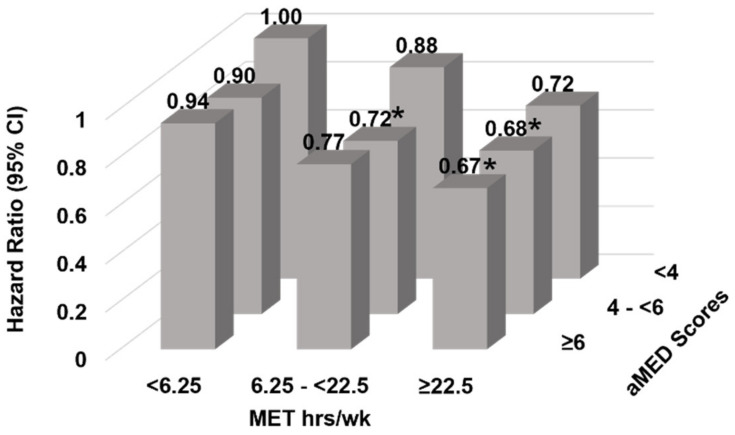
Adjusted Hazard ratios (95% CI) of CVD mortality for the joint association between pre-cancer diagnosis Alternate Mediterranean Diet score and metabolic equivalent task hours per week score among female cancer survivors in the California Teachers Study cohort (*n* = 18,533). Adjusted for interval from questionnaire 1 to cancer diagnosis date, age at cancer diagnosis, race, smoking status, menopausal status and hormone use, total energy intake, and daily vitamin use. * Signifies significant difference (*p* < 0.05).

Despite no significant interaction between pre-cancer diagnosis aMED and PA in relation to CVD mortality, the joint association of diet and PA showed lower CVD mortality risk among those who had higher PA levels and aMED scores (Figure 1). Compared to those with the lowest levels of pre-diagnosis aMED scores and PA, cancer survivors with the highest levels of PA had 32% and 33% lower risk of CVD mortality when their aMED score was ≥6 (HR = 0.67; 95% CI: 0.52–0.87) or between 4 and 6, respectively; HR = 0.68 (0.52, 0.90). (Supplemental Table S2; Model 2: P_interaction_ = 0.13). 

Sensitivity analyses to account for post-diagnosis aMED scores and PA was conducted (Table 3). These associations with CVD mortality risk were no longer significant after adjusting for post-cancer diagnosis data. This may be due to a smaller sample size and converting to tertiles because of small sample size. Post hoc power analysis showed 50% and 76% power to detect a significant association of the aMED and PA level with CVD mortality, respectively. 

## 4. Discussion

Previous research has shown an increased risk of CVD in cancer survivors compared to the general population [3,4]. With this increased risk of CVD, and a parallel growth in cancer survivors, the importance of lifestyle factors before cancer diagnosis in assessing risk of CVD mortality in cancer survivors helps inform clinicians in cardio-oncology. This secondary analysis investigated the association between pre-cancer diagnosis diet quality and PA level and their interaction on CVD mortality in female cancer survivors. We did not find a significant association between aMED score and CVD mortality, but we did find a significant inverse relationship between leisure-time PA level prior to cancer diagnosis and CVD mortality in this population. We observed that regardless of the aMED score, a higher PA activity level was a stronger predictor for CVD mortality.

When looking at the characteristics of this population based on the aMED score, higher scores were associated with older participants. A possible explanation for this is having more time available to focus on eating habits in lieu of parenthood and job responsibilities. If individuals were older when they participated and had no history of cancer diagnosis, they would also be older when they were diagnosed with cancer. Individuals with higher aMED scores could also be more health conscious, which may further explain their lower BMIs and higher PA levels. Higher energy intake was also associated with higher aMED scores, which may seem counter-intuitive because higher aMED scores were associated with lower BMI; however, the highest energy intake quartile was, on average, still below 2000 kilocalories, and these participants also had higher PA levels which may balance energy intake and expenditure.

Previous work has shown an inverse association between diet quality and all-cause mortality in cancer survivors [16,17,34,35]. These studies did not explore the specific relationship between pre-diagnosis diet quality and CVD mortality in cancer survivors. To our knowledge, this relationship has not been investigated in this cohort. We assessed the risk of death from CVD based on the aMED score in this population and found no significant association between the pre-cancer diagnosis aMED score and CVD mortality risk. It is possible that a different dietary index would be a stronger predictor of CVD mortality in this population, such as the Alternative Healthy Eating Index (AHEI) which has been shown to decrease CVD mortality risk [36]. However, because the amount of dietary *trans* fat and omega-3 fatty acids were not available for the CTS cohort, we could not calculate AHEI scores.

We also estimated CVD mortality risk based on pre-diagnosis PA and found an inverse association between pre-diagnosis PA level and CVD mortality. PA was associated with up to 20% lower CVD mortality risk for participants with PA levels of 27.5 MET hrs/wk or more. A total of 27.5 MET hrs/wk is equivalent to running (~9 METs) for an hour 3 times a week or taking a walk (~4 METs) for an hour every day of the week. Lower CVD mortality risk was also seen among participants with PA levels ranging between 13.5 MET hrs/wk and 27.5 MET hrs/wk. For example, 15 MET hrs/wk could be attained with moderate-intensity exercise (~5 METs) for 3 times a week. Thus, independent of aMED score, a PA level approximately twice the current PA recommendations (7.5 MET hrs/wk), prior to cancer diagnosis, could reduce CVD mortality risk in cancer survivors. Previous work has reported post-cancer diagnosis adherence to the American Cancer Society PA guidelines (7.5 MET hrs/wk) was associated with lower risk of CVD mortality among cancer survivors [37].

Since PA and diet are important lifestyle behaviors related to CVD mortality, we tested their interaction and no statistical significance was observed. However, a higher PA level was associated with lower CVD mortality risk in both aMED score categories (4-<6 and ≥6). There is very little research on the interaction between diet quality and PA on CVD mortality in cancer survivors. One study showed that higher post-cancer diagnosis aMED scores were associated with lower risk of non-breast cancer mortality in women with low PA [18], but CVD mortality was not investigated among these cancer patients.

A strength of our study is the mitigation of reverse causality bias. Cancer survivors can experience many symptoms, such as fatigue and nausea, which may affect diet quality and PA level [38,39]. To avoid biased estimates of the changes of PA levels from before to after cancer diagnosis that could be due to disease symptoms and its severity, questionnaires collected after a year from cancer diagnosis were only included. Unfortunately, we did not have enough power to adequately address this concern; however, it appears that adjusting for post-diagnosis PA level made the associations between pre-cancer diagnosis PA level and risk for CVD mortality weaker.

There are some limitations to our study. The first is that only one dietary index was used and portion sizes for the FFQ in questionnaire 1 and in questionnaire 4 were different. Questionnaire 1 FFQ used small, medium, and large portions, whereas questionnaire 4 FFQ was based on pictures of cup sizes for participants to determine their portions. Moreover, there was a ten-year difference between these questionnaires. Thus, changes in reporting of portion sizes is reflective of changes in cultural norms related to portion size [40]. However, aMED scores were calculated separately in each questionnaire cycle, and participants were then ranked to each other. Another limitation is that we did not have enough statistical power to stratify associations by cancer type. When stratified by cancer type, power dropped down to 40% for diet and 73% for PA. Given the limited power for cancer type, we could not additionally stratify CVD mortality risk based on cancer treatment. This is a limitation, as there are several cancer types for which there are many different treatment options, with some having higher risk for CVD mortality [41].

## 5. Conclusions

This is the first study to examine the relationship between diet quality and PA prior to cancer diagnosis and their interaction on CVD mortality in female cancer survivors. Research in this area is scarce and more work is needed to confirm the relationship between pre-diagnosis diet quality and PA and their interaction on CVD mortality in other populations. Overall, this study showed an inverse association between pre-cancer diagnosis PA level and CVD mortality in female cancer survivors. This relationship was no longer significant when adjusting for post-diagnosis PA level (however, this model was underpowered). Nonetheless, our observations support the importance of PA throughout the lifecycle in decreasing risk of CVD mortality. Primary care physicians (PCPs) should continue to relay the significance of PA level in their healthy patients prior to a potential diagnosis of cancer and follow the recommendations of the AHA and ACS. Additionally, it is important for clinicians to recognize that a cancer survivor who had high PA levels prior to their cancer diagnosis may have decreased risk for CVD mortality during cancer survivorship.

## Figures and Tables

**Table 1:** Age adjusted ^1^ characteristics of female cancer survivors in the California Teachers Study cohort by quintiles of the pre-cancer diagnosis Alternative Mediterranean Diet score (*n* = 18,533 participants).

Characteristics	Overall *n* = 18,533	aMED				
		Q1	Q2	Q3	Q4	Q5
aMED score, mean ± SD	4.7 ± 1.8	2.4 ± 0.8	4.0 ± 0.0	5.0 ± 0.0	6.0 ± 0.0	7.4 ± 0.6
Physical activity, MET-hrs/wk mean ± SD	20.0 ± 23.1	14.7 ± 19.9	18.3 ± 21.7	20.6 ± 23.3	22.7 ± 23.8	26.6 ± 26.1
Age, yr mean ± SD	56.2 ± 12.0	53.1 ± 11.9	55.4 ± 11.8	56.8 ± 11.9	58.1 ± 11.9	59.1 ± 11.3
Years of diagnosis, *n* (%)						
<5 yrs	8099 (43.7)	2169 (44.9)	1512 (44.6)	1605 (43.8)	1427 (43.2)	1386 (41.7)
5-<10 yrs	4347 (23.5)	1143 (22.6)	826 (24.0)	868 (24.0)	732 (22.7)	778 (24.1)
≥10 yrs	6087 (32.8)	1649 (32.6)	1078 (31.3)	1165 (32.2)	1097 (34.1)	1098 (34.1)
Age of diagnosis, yr mean ± SD	70.0 ± 11.9	65.0 ± 11.9	67.1 ± 11.8	68.3 ± 12.0	69.4 ± 11.9	70.3 ± 11.5
Baseline BMI, *n*(%)						
<18 kg/m^2^	874 (4.7)	217 (4.8)	155 (4.7)	189 (5.1)	166 (4.9)	147 (4.1)
18-<22.5 kg/m^2^	5957 (32.1)	1563 (30.3)	1047 (30.3)	1122 (31.1)	1065 (33.6)	1160 (37.3)
22.5-<25 kg/m^2^	4092 (22.1)	1010 (20.2)	743 (21.9)	804 (22.1)	730 (22.2)	805 (24.3)
25-<30 kg/m^2^	4827 (26.1)	1260 (26.2)	918 (27.0)	994 (27.1)	851 (25.6)	804 (23.6)
≥30 kg/m^2^	2783 (15.0)	911 (18.5)	553 (16.1)	529 (14.6)	444 (13.7)	346 (10.7)
Alcohol intake, %						
Never drinker	5739 (31.0)	1969 (39.8)	1148 (33.7)	1135 (31.1)	872 (26.5)	615 (18.3)
<15 g/day	9089 (49.0)	2109 (41.0)	1600 (46.5)	1795 (49.6)	1667 (51.9)	1918 (60.2)
≥15 g/day	3705 (20.0)	883 (19.2)	668 (19.8)	708 (19.3)	717 (21.6)	729 (21.5)
Race/ethnicity, %						
White	16,649 (89.8)	4354 (88.6)	3071 (90.0)	3294 (90.5)	2946 (90.1)	2984 (91.2)
Other race/unspecified	1884 (10.2)	607 (11.4)	345 (10.0)	344 (9.5)	310 (9.9)	278 (8.8)
Menopausal status and HT use, %						
Premenopausal	5181 (30.0)	1801 (28.2)	1038 (28.8)	951 (27.7)	743 (27.3)	648 (27.3)
Peri-/postmenopausal, never use HT	2587 (14.0)	654 (16.1)	434 (13.3)	553 (14.7)	479 (13.3)	467 (12.4)
Peri-/postmenopausal, past use HT	1442 (7.8)	303 (7.7)	248 (7.7)	311 (8.2)	290 (7.9)	290 (7.4)
Peri-/postmenopausal, current use HT	6433 (34.7)	1443 (32.7)	1136 (34.0)	1272 (34.1)	1228 (35.4)	1354 (36.9)
Peri-/postmenopausal, unknown HT use	902 (4.9)	196 (5.0)	188 (5.7)	171 (4.5)	165 (4.6)	182 (4.8)
Unknown menopausal status	1988 (10.7)	564 (10.4)	372 (10.4)	380 (10.7)	351 (11.5)	321 (11.2)
Smoking status, %						
Smokers	7349 (39.6)	1858 (39.8)	1363 (40.2)	1459 (39.8)	1303 (39.3)	1366 (40.2)
Non-smokers/unknown	11,184 (60.4)	3103 (60.2)	2053 (59.8)	2179 (60.2)	1953 (60.7)	1896 (59.8)
History of diabetes, %	478 (2.6)	134 (3.0)	84 (2.55)	99 (2.7)	88 (2.6)	73 (2.0)
History of hypertension, %	3587 (19.4)	938 (21.5)	648 (19.5)	715 (19.2)	657 (18.8)	639 (17.3)
Daily vitamin use, %	12,474 (67.3)	2909 (60.3)	2241 (65.9)	2473 (67.7)	2331 (71.1)	2520 (76.3)
Total energy intake, kcal/d mean ± SD	1561.6 ± 499.6	1382.2 ± 454.4	1519.1 ± 489.7	1566.2 ± 497.0	1687.2 ± 516.6	1762.7 ± 487.0

^1^ All variables except for age and age of diagnosis are age-standardized. aMED = Alternative Mediterranean Diet, SD = standard deviation, Q = quintile, BMI = body mass index, HT = hormonal therapy.

**Table 2:** Hazard ratio (95% CIs) of CVD mortality associated with pre-cancer diagnosis Alternative Mediterranean Diet scores and physical activity levels among female cancer survivors in the California Teachers Study cohort (*n* = 18,533).

aMED, Quintiles (Score Range)	Cases	Person-Years	Model 1	Model 2	Model 3
Q1 (<4)	198	37,193	1.00 (reference)	1.00 (reference)	1.00 (reference)
Q2 (4-<5)	147	25,153	0.87 (0.71, 1.08)	0.87 (0.70, 1.08)	0.88 (0.71, 1.09)
Q3 (5-<6)	179	26,986	0.86 (0.71, 1.06)	0.87 (0.71, 1.06)	0.89 (0.73, 1.10)
Q4 (6-<7)	200	24,632	0.90 (0.72, 1.10)	0.90 (0.71, 1.10)	0.93 (0.76, 1.15)
Q5 (≥7)	191	24,778	0.84 (0.69, 1.03)	0.83 (0.67, 1.03)	0.89 (0.72, 1.11)
			P_trend_ = 0.16	P_trend_ = 0.16	P_trend_ = 0.47
**Physical activity in recent 3 years prior to baseline, MET-hrs/wk,** **Quartiles**	**Cases**	**Person-Years**	**Model 1**	**Model 2**	**Model 4**
Q1 (<3.5)	292	34,162	1.00 (reference)	1.00 (reference)	1.00 (reference)
Q2 (3.5-<13.5)	207	34,204	0.86 (0.72, 1.02)	0.86 (0.72, 1.03)	0.87 (0.72, 1.04)
Q3 (13.5-<27.5)	207	34,177	0.77 (0.65, 0.93)	0.79 (0.66, 0.94)	0.80 (0.67, 0.96)
Q4 (≥27.5)	209	36,199	0.73 (0.61, 0.87)	0.73 (0.61, 0.87)	0.74 (0.61, 0.88)
			P_trend_ < 0.001	P_trend_ < 0.001	P_trend_ = 0.0014

Model 1: Adjusted for time interval from questionnaire 1 to cancer diagnosis date, age at cancer diagnosis. Model 2: Adjusted for time interval from questionnaire 1 to cancer diagnosis date, age at cancer diagnosis, race, smoking status, menopausal status and hormone use, total energy intake, and daily vitamin use. Model 3: Adjusted for physical activity + covariates included in Model 2. Model 4: Adjusted for aMED + covariates included in Model 2. CI = confidence interval, CVD = cardiovascular disease, aMED = Alternative Mediterranean Diet, Q = quintile for diet and quartile for physical activity.

**Table 3:** Hazard ratios (95% CIs) of CVD mortality associated with pre-cancer diagnosis Alternative Mediterranean Diet scores and physical activity levels after adjustment for post-diagnosis levels among female cancer survivors with available data at least 1 year after cancer diagnosis in the California Teachers Study cohort (*n* = 3327).

aMED, Tertiles	Cases	Person-Years	Model 1	Model 2	Model 3
<4	39	12,671	1.00 (reference)	1.00 (reference)	1.00 (reference)
4-<6	56	17,985	0.76 (0.50, 1.16)	0.78 (0.51, 1.19)	0.83 (0.54, 1.28)
≥6	86	17,869	0.84 (0.56, 1.26)	0.87 (0.58, 1.33)	1.07 (0.69, 1.65)
			P_trend_ = 0.54	P_trend_ = 0.68	P_trend_ = 0.62
**Physical activity in recent 3 years prior to baseline, MET-hrs/wk,** **Tertiles**	**Cases**	**Person-Years**	**Model 1**	**Model 4**	**Model 5**
<6.25	74	15,365	1.00 (reference)	1.00 (reference)	1.00 (reference)
6.25-<22.5	48	15,724	0.74 (0.51, 1.08)	0.76 (0.52, 1.10)	0.76 (0.52, 1.11)
≥22.5	59	17,426	0.79 (0.56, 1.12)	0.83 (0.57, 1.19)	0.84 (0.56, 1.25)
			P_trend_ = 0.25	P_trend_ = 0.38	P_trend_ = 0.44

Model 1: Adjusted for interval from questionnaire 1 to cancer diagnosis date, age at cancer diagnosis, race, smoking status, menopausal status and hormone use, total energy intake, and daily vitamin use. Model 2: Additionally adjusted for physical activity before cancer diagnosis. Model 3: Additionally adjusted for physical activity before cancer diagnosis + aMED after cancer diagnosis. Model 4: Additionally adjusted for aMED before cancer diagnosis. Model 5: Additionally adjusted for aMED before cancer diagnosis + physical activity after diagnosis. CI = confidence interval, CVD = cardiovascular disease, aMED = Alternative Mediterranean Diet.

## Data Availability

The datasets analyzed for this study can be found in the California Teachers Study: https://www.calteachersstudy.org/cts-data (accessed on 15 February 2022).

## Supplementary Materials

The following supporting information can be downloaded at: https://www.mdpi.com/article/10.3390/cancers14133096/s1, Table S1: Age adjusted characteristics of female cancer survivors in the California Teachers Study cohort by quartiles of the pre-cancer diagnosis Metabolic Equivalent Task Scores; Table S2: HRs (95% CIs) for association between aMED, physical activity, and CVD death.
