# Supplementary material for: Pre-Diagnosis Diet and Physical Activity and Risk of Cardiovascular Disease Mortality among Female Cancer Survivors
Source: Cancers (Basel). 2022 Jun 23;14(13):3096. doi: 10.3390/cancers14133096 (PMC9265097; doi:10.3390/cancers14133096)
Supplement: Supplementary file 1 [file cancers-14-03096-s001.zip › cancers-1737858-supplementary.pdf]

cancers

MDPI

Supplementary Materials

Pre-Diagnosis Diet and Physical Activity and Risk of Cardiovascular Disease Mortality among Female Cancer Survivors

Table S1. Age adjusted[1] characteristics of female cancer survivors in the California Teachers Study cohort by quartiles of the pre-cancer diagnosis Metabolic Equivalent Task Scores (N=18,533 participants).

Characteristics	Physical Activity in 3 Years Prior to Baseline			
	Q1	Q2	Q3	Q4
**aMED score, mean ± SD**	4.1 ± 1.8	4.6 ±1.8	4.9 ± 1.8	5.2 ± 1.8
**Physical activity, MET-hrs/wk mean ± SD**	0.9 ± 1.2	7.5 ± 2.5	19.1 ± 4.2	51.2 ± 24.0
**Age, yr mean ± SD**	56.9 ± 12.1	55.5 ± 11.9	56.5 ± 11.8	55.8 ± 12.1
**Years of diagnosis, n (%)**				
**>0- <5 yrs**	2,057 (44.2)	1,994 (43.8)	2,029 (44.2)	2,019 (42.5)
**5 - <10 yrs**	1,047 (23.0)	1,081 (23.5)	1,073 (23.5)	1,146 (24.1)
**≥ 10 yrs**	1,508 (32.8)	1,506 (32.7)	1,486 (32.4)	1,587 (33.4)
**Age of diagnosis, yr mean ± SD**	68.4 ± 11.8	67.4 ± 11.8	68.4 ± 11.8	67.7 ± 12.2
**Baseline BMI, n(%)**				
**< 18 kg/m²**	224 (4.7)	201 (4.5)	219 (4.8)	230 (4.8)
**18 - <22.5 kg/m²**	1,155 (25.3)	1,321 (28.6)	1,539 (33.7)	1,942 (41.0)
**22.5 - <25 kg/m²**	840 (18.2)	970 (21.2)	1,093 (23.8)	1,189 (25.0)
**25 - <30 kg/m²**	1,309 (28.3)	1,279 (28.1)	1,190 (25.8)	1,049 (22.0)
**≥ 30 kg/m²**	1,084 (23.5)	810 (17.5)	547 (11.9)	342 (7.1)
**Alcohol intake, %**				
**Nondrinker**	1,767 (38.2)	1,469 (32.2)	1,279 (27.9)	1,224 (25.7)
**< 15 g/day**	2,090 (45.7)	2,287 (49.6)	2,358 (51.4)	2,358 (49.6)
**≥ 15 g/day**	755 (16.1)	825 (18.2)	955 (20.7)	1,170 (24.8)
**Race/ethnicity, %**				
**White**	4,001 (86.4)	4,107 (89.7)	4,189 (91.2)	4,352 (91.8)
**Other Race/unspecified**	611 (13.6)	474 (10.3)	399 (8.8)	400 (8.2)
**Menopausal status and HT use, %**				
**Premenopausal**	1,199 (28.2)	1,367 (27.8)	1,242 (27.9)	1,373 (28.0)
**Peri-/postmenopausal, never use HT**	716 (15.0)	611 (14.1)	616 (13.1)	644 (13.7)
**Peri-/postmenopausal, past use HT**	404 (8.5)	335 (7.7)	367 (7.8)	336 (7.1)
**Peri-/postmenopausal, current use HT**	1,536 (32.6)	1,533 (34.7)	1,651 (35.5)	1,713 (36.0)
**Peri-/postmenopausal, unknow HT use**	240 (5.0)	201 (4.6)	238 (5.1)	223 (4.7)
**Unknown menopausal status**	517 (10.8)	534 (11.1)	474 (10.5)	463 (10.5)
**Smoking status, %**				
**Smokers**	1,764 (37.8)	1,757 (38.7)	1,818 (39.4)	2,010 (42.8)
**Non-Smokers/unknown**	2,848 (62.2)	2,824 (61.3)	2,770 (60.6)	2,742 (57.2)
**History of diabetes, %**	156 (3.3)	125 (2.8)	108 (2.3)	89 (1.9)
**History of hypertension, %**	1,091 (23.0)	908 (20.5)	827 (17.9)	771 (16.3)
**Daily Vitamin use, %**	2,814 (60.7)	3,040 (66.8)	3,170 (68.9)	3,450 (72.8)
**Total energy intake, kcal/d mean ± SD**	1,561.6 ± 523.0	1,572.5 ± 497.8	1,548.0 ± 476.0	1,565.2 ± 501.3

aMED=alternative Mediterranean diet, SD=standard deviation, Q=quartile, BMI=body mass index, HT=hormonal therapy [1] All variables except for age and age of diagnosis are age-standardized.

Table S2. HRs (95% CIs) for association between aMED, physical activity, and CVD death.

aMED Score, Tertiles	Physical Activity, MET-hrs/wk	Cases	Person-Years	HR (95% CI)[1]	HR (95% CI)[2]
**<4**	**<6.25**	101	16,542	1.00 (reference)	1.00 (reference)
	**6.25 - <22.5**	52	11,556	0.89 (0.64, 1.24)	0.88 (0.63, 1.24)
	**≥22.5**	45	9,095	0.72 (0.50, 1.02)	0.72 (0.51, 1.03)
**4 - <6**	**<6.25**	140	17,255	0.92 (0.71, 1.19)	0.90 (0.69, 1.17)
	**6.25 - <22.5**	89	16,523	0.71 (0.53, 0.94)	0.72 (0.54, 0.96)
	**≥22.5**	97	18,361	0.68 (0.51, 0.89)	0.68 (0.52, 0.90)
**≥6**	**<6.25**	132	11,501	0.94 (0.72, 1.21)	0.94 (0.72, 1.23)
	**6.25 - <22.5**	113	16,339	0.77 (0.59, 1.01)	0.77 (0.59, 1.01)
	**≥22.5**	146	21,569	0.67 (0.53, 0.87)	0.67 (0.52, 0.87)
				$P_{interaction}$=0.12	$P_{interaction}$=0.13

HR=hazard ratio, CI=confidence interval, CVD=cardiovascular disease, aMED =alternative Mediterranean diet. [1] Adjusted for interval from questionnaire 1 to cancer diagnosis, age at cancer diagnosis (Model 1). [2] Adjusted for time interval from questionnaire 1 to cancer diagnosis date, age at cancer diagnosis, race, smoking status, menopausal status and hormone use, total energy intake, and daily vitamin use (Model 2).
